# Development and reliability of questionnaires for the assessment of diet and physical activity behaviors in a multi-country sample in Europe the Feel4Diabetes Study

**DOI:** 10.1186/s12902-019-0469-x

**Published:** 2020-03-12

**Authors:** Costas A. Anastasiou, Evaggelia Fappa, Konstantina Zachari, Christina Mavrogianni, Vicky Van Stappen, Jemina Kivelä, Eeva Virtanen, Esther M. González-Gil, Paloma Flores-Barrantes, Anna Nánási, Csilla Semánová, Roumyana Dimova, Natalya Usheva, Violeta Iotova, Greet Cardon, Yannis Manios, Konstantinos Makrilakis, Feel4Diabetes-study group, Jaana Lindström, Peter Schwarz, Lieven Annemans, Ignacio Garamendi, Meropi Kontogianni, Odysseas Androutsos, Konstantina Tsoutsoulopoulou, Christina Katsarou, Eva Karaglani, Irini Qira, Efstathios Skoufas, Konstantina Maragkopoulou, Antigone Tsiafitsa, Irini Sotiropoulou, Michalis Tsolakos, Effie Argyri, Mary Nikolaou, Eleni-Anna Vampouli, Christina Filippou, Katerina Gatsiou, Efstratios Dimitriadis, Tiina Laatikainen, Katja Wikström, Jemina Kivelä, Päivi Valve, Esko Levälahti, Greet Cardon, Vicky Van Stappen, Nele Huys, Ruben Willems, Samyah Shadid, Peter Schwarz, Ivonne Panchyrz, Maxi Holland, Patrick Timpel, Stavros Liatis, George Dafoulas, Christina-Paulina Lambrinou, Angeliki Giannopoulou, Lydia Tsirigoti, Evi Fappa, Costas Anastasiou, Lala Rabemananjara, Maria Stella de Sabata, Winne Ko, Luis Moreno, Fernando Civeira, Gloria Bueno, Pilar De Miguel-Etayo, Esther M. Gonzalez-Gil, Maria I. Mesana, Germán Vicente-Rodriguez, Gerardo Rodriguez, Lucia Baila-Rueda, Ana Cenarro, Estíbaliz Jarauta, Rocío Mateo-Gallego, Violeta Iotova, Tsvetalina Tankova, Natalia Usheva, Kaloyan Tsochev, Nevena Chakarova, Sonya Galcheva, Rumyana Dimova, Yana Bocheva, Zhaneta Radkova, Vanya Marinova, Yuliya Bazdarska, Tanya Stefanova, Imre Rurik, Timea Ungvari, Zoltán Jancsó, Anna Nánási, László Kolozsvári, Csilla Semánova, Remberto Martinez, Marcos Tong, Kaisla Joutsenniemi, Katrina Wendel-Mitoraj

**Affiliations:** 1 Department of Nutrition and Dietetics, School of Health Science and Education, Harokopio University, Athens, Greece; 2 First Department of Propaedeutic Internal Medicine, Medical School, National and Kapodistrian University of Athens, Athens, Greece; 3 Department of Movement and Sports Sciences, Faculty of Medicine and Health Sciences, Ghent University, Ghent, Belgium; 4 Department of Public Health Solutions, National Institute for Health and Welfare, Helsinki, Finland; 5 GENUD (Growth, Exercise, Nutrition and Development) Research Group, Faculty of Health Sciences, University of Zaragoza, Zaragoza, Spain; 6 Instituto Agroalimentario de Aragón (IA2), Zaragoza, Spain; 7 Instituto de Investigación Sanitaria Aragón (IIS Aragón), Zaragoza, Spain; 8 Centro de Investigación Biomédica en Red de Fisiopatología de la Obesidad y Nutrición (CIBERObn), Madrid, Spain; 9 Department of Family and Occupational Medicine, Faculty of Public Health, University of Debrecen, Debrecen, Hungary; 10 Department of Diabetology, Clinical, Center of Endocrinology, Medical University of Sofia, Sofia, Bulgaria; 11 Department of Social Sciences and Public Health, Medical University of Varna, Varna, Bulgaria; 12 Department of Pediatrics, Clinic of Pediatric Endocrinology, Medical University of Varna, Varna, Bulgaria; 13 Department of Internal Medicine, National and Kapodistrian University of Athens Medical School, Laiko General Hospital, 17 Ag. Thoma St, 11527 Athens, Greece

**Keywords:** Diet, Energy balance behaviors, Sedentary lifestyle, Nutrition, Physical activity, Reliability, Questionnaire

## Abstract

**Background:**

Assessment of diet and physical activity and their determinants still remains a demanding task, especially when the objective is to evaluate the efficacy of lifestyle interventions. In the context of the Feel4Diabetes study (a European community based intervention study in families with school aged children and at high risk of developing diabetes), we aimed to develop questionnaires for the assessment of food-frequency and eating behaviors, and physical activity and sedentary behaviors in both parents and school-aged children and a questionnaire for overall family’s energy balance-related behaviors.

**Methods:**

Questionnaires were developed to be used in 6 countries under standardized harmonization procedures and included questions regarding not only food intake and physical activity, but also questions of their determinants. A reliability study was conducted in 191 pairs of parents and their children (*N* = 191). Parents completed the questionnaires on two occasions, within a 1–2 week interval. Reliability was tested by the intra-class correlation coefficients (ICC) of test-retest.

**Results:**

Most of the questions in all questionnaires had excellent reliability, assessed as an ICC of > 0.810. Mean ICCs for food-frequency and eating behaviors questionnaires were 0.838 and 0.787, and for physical activity and sedentary behaviors questionnaires were 0.734 and 0.793, in adults and children respectively. Mean ICC for overall family’s energy balance-related behaviors and their determinants was 0.659.

**Conclusion:**

The developed questionnaires showed acceptable reliability and may be valuable tools in the assessment of children’s and parents’ behaviors related to diet, physical activity, sedentary behavior and overall energy balance in school- and community-based interventions.

## Background

Diet and physical activity are key lifestyle factors to improve overall health, including diabetes prevention and enhancement of glycemic control throughout the lifespan. A recent review of clinical trials suggests that both diet and physical activity interventions are capable to reduce the risk of type 2 diabetes, especially in the high risk population, with their combinations being more potent [1]. In school-aged children, interventions for prudent eating habits [2] and increased levels of physical activity [3] have been also associated with increased insulin sensitivity after a 2 years follow up.

Diet and physical activity have qualitative and quantitative aspects and their precise assessment is of major importance in identifying those at risk, in developing targeted interventions and also in measuring the efficacy of these interventions. Unfortunately, all methods of measuring dietary intake are hampered by errors of precision [4]. Assessments of intakes of specific nutrients obtained with a food frequency questionnaire, in comparison to intake with a gold standard method, have yielded low correlation coefficients in both adults [5] and children [6]. It has been proposed that measuring consumption of food groups and their integration into dietary patterns, rather than assessment of single nutrients intake, may be more closely associated to various health outcomes, due to the highly interrelated nature of dietary exposures [7]. However, reported correlations between actual and measured intakes of specific food groups have been also weak to moderate in children [8]. The same constraints apply when assessing physical activity levels in various age groups [9, 10].

A relatively new era of research suggests that food choice and physical activity are influenced by multiple environmental factors that may augment or diminish a healthy behavior [11]. At the individual level, it has been observed that many healthy or unhealthy behaviors do not occur by chance, but rather they cluster [12] and may be interrelated. For example, screen time has been associated with poor eating habits in children and adolescents [13]. Delineating these complex interactions and their environmental determinants is of critical importance in order to target specific diet and physical activity behaviors that can be modified by behavioral change based interventions. Unfortunately, there is scarcity of well-designed tools for the assessment of such aspects of diet and physical activity at the community level.

In this paper we describe the development of questionnaires for diet and physical activity assessment in adults and school-aged children in the context of the Feel4Diabetes Study. The study regards a school- and community-based intervention in 6 European countries, aiming to promote a healthy lifestyle and tackle obesity and obesity-related metabolic factors related to increased risk for type 2 diabetes. We aimed to develop questionnaires capable in assessing not only dietary intake and physical activity status, but also overall energy balance-related behaviors, perceptions and environmental barriers or facilitators for each behavior.

## Methods

### Setting

The Feel4Diabetes study aims to develop, implement and evaluate a school- and community-based intervention to prevent type 2 diabetes among families from low- and middle-income countries and vulnerable populations in high-income countries in Europe (http://feel4diabetes-study.eu). The intervention is applied in six European countries (Belgium, Bulgaria, Finland, Greece, Hungary and Spain) and targets dietary and physical activity behaviors. Participating families included at least one parent/care giver and one child attending one of the first three grades of compulsory education. The research group of the study includes medical doctors, researchers in the area of nutrition and exercise (with a Master’s or a Doctorate degree), as well as practitioners in the field (with at least a Bachelor’s degree). The members of the research group were both males and females and all were adequately trained for the purposes of the study. It is of great importance that multicenter studies use harmonized and standardized measurement procedures as well as reliable and valid tools to assess the effectiveness of intervention programs [14].

### Development of questionnaires

We developed a total of 5 questionnaires: two food-frequency and eating behaviors questionnaires (one for adults and one for children), two physical activity and sedentary behaviors questionnaires (one for adults and one for children) and a brief questionnaire assessing overall family’s energy balance related behaviors and their determinants. Development of each questionnaire was based on previous experiences from similar studies, by adding questions in order to also assess determinants of each behavior, based on the relevant bibliography. Questionnaires were designed to be self-administered and be answered by the parent/caregiver of each family. We limited open-ended questions as much as possible and we applied odd numbers in response options to close-labeled questions.

The initial forms of the questionnaires were developed in the English language and were commented and approved by all study partners. To ensure quality and consistency across local versions, the developed questionnaires were forward - translated in the language of each participating country, culturally adapted and back-translated [15]. If any discrepancies, uncertainties or mistakes were identified, appropriate corrections were made until the highest standard of precision in the translation was reached.

#### Food-frequency and eating behaviors questionnaires

Food-frequency questions in the questionnaire for adults were derived from a questionnaire developed for the National Type 2 diabetes prevention program in Finland (FIN-D2D) [16], with some modifications so as to be relevant for the target multi-country population of the Feel4Diabetes study. A portion size defined with a household unit was provided for foods under question and the available answers provided frequency of consumption of the specified portion of each food. Additional questions were entered regarding meals and snacks consumption during weekdays and weekend days, consumption of specific foods during breakfast, and participant’s perception of body weight and knowledge for adequate consumption of fruits and vegetables. The questionnaire for children was similar with that developed for adults, with minor exceptions, for example without questions regarding coffee or alcohol consumption.

#### Physical activity and sedentary behaviors questionnaires

Physical activity questions in the questionnaire for adults were based on the short form of the International Physical Activity Questionnaire [17]. We added more detailed questions on sedentary behaviors, including time spending on TV viewing, using a computer/laptop/smartphone on weekdays and weekend days. We also added questions regarding social support, perceptions and attitudes for physical activity, environmental determinants and knowledge about physical activity recommendations for adults. The physical activity questionnaire for children was initially based on a relevant questionnaire that was developed for the needs of a previous school-based intervention of our research group (the ToyBox study [18]), with major modifications in order to be applicable in school-aged children. The same principles were used, as in the case of the adults’ questionnaire.

#### Family’s energy balance related behaviors questionnaire

A separate questionnaire was developed in order to assess habits related to diet (frequency of consumption of breakfast and of consumption of key food groups) and physical activity (total time of physical activity and screen activities excluding work/school) for the parent/caregiver and the child. A set of questions examined also availability of healthy or unhealthy food choices at home, parenting practices that could influence children’s eating behavior related to diet and physical activity and sedentary behavior, perceptions regarding health and availability of electronic devices in the child’s room.

### Reliability of questionnaires

Reliability addressed the question of how consistent the answers were from one occasion to the next in the same subject in terms of physical activity, sedentary behavior, food and beverage consumption and eating behavior. The study sample comprised of parents/primary caregivers from the six countries of the study who had similar demographic characteristics with the targeted population in the Feel4Diabetes-intervention (i.e. children attending the first three grades in primary school; families living in low socioeconomic status municipalities in Finland, Belgium, Greece and Spain or the overall population in Bulgaria and Hungary). Parents/caregivers were asked to complete the questionnaires twice, within a 1–2 week interval. Parents/caregivers were contacted through their children’s school and an information letter regarding the objectives of the study and instructions on how to complete the questionnaires was sent to them. Written consents were obtained. Since this is a validation study of the developed questionnaires, participation rate and reasons not to participate were not recorded. One researcher in each country performed data processing and participant’s coding.

The agreement between categorical, continuous and dichotomous items was analyzed using a two-way random effect single measure intra-class correlation coefficient (ICC). ICCs were classified as “excellent” (> 0.81), “good” (0.61–0.80), “moderate” (0.41–0.60) or “poor” (< 0.40). Additional analyses were performed in order to check for differences between countries, using one-way analysis of variance. Data processing and analyses were performed with IBM SPSS Statistics, Version 24, Armonk, NY, IBM Corporation.

## Results

The developed questionnaires are available upon request, either in English or in one of the other six available languages (Bulgarian, Finnish, Flemish, Greek, Hungarian or Spanish).

### Reliability results

A total of 191 pairs of parents/caregivers and their children from the six countries of the study participated in the reliability study of the questionnaires (Ν = 35, 32, 20, 11, 30 and 63 for Belgium, Bulgaria, Finland, Greece, Hungary and Spain, respectively). Mean age of the study participants was 41.0 ± 5.4 and 9.1 ± 1.8 years respectively. 83% were mothers and 17% fathers. 19% of the participating parents had total years of education less than 12 years. No significant differences were observed in reliability results between countries, thus results are presented for the total sample of the study.

#### Reliability of food-frequency and eating behaviors questionnaires

ICCs for food-frequency and eating behaviors questions ranged from 0.238 to 0.956 in adults and from 0.349–0.953 in children (Table 1 and Additional files 1 and 2: Tables S1 and S2). Overall, in adults’ questionnaire, 77.4% of ICCs were ranked as “excellent”, 20.2% as “good” and 2.4% as poor. In children’s questionnaire, 64.1% of ICCs were ranked as “excellent”, 30.8% as “good”, 2.6% as “moderate” and 2.5% as “poor”. In adults, with the exception of the question on nutrition knowledge, where relatively low reliability was observed, all other categories of questions showed similar reliability. In children, the question about breakfast skipping had relatively lower reliability, compared to other categories. Food-frequency questions had lower ICCs in children, compared to adults. Nutrition knowledge of the parents for children seemed to be higher than nutrition knowledge for adults.

**Table 1:** Summary of reliability measures of food-frequency and eating behaviors questions in adults and children

	Adults		Children	
	Number of questions	Mean ICC (range)	Number of questions	Mean ICC (range)
Frequency of consumption of specific foods	33	0.832 (0.349–0.947)	29	0.767 (0.349–0.953)
Types of fat consumed	15	0.862 (0.786–0.936)	15	0.807 (0.484–0.939)
Meals and snacks frequency	14	0.819 (0.238–0.956)	14	0.851 (0.778–0.925)
Foods consumption at breakfast	16	0.848 (0.667–0.924)	15	0.789 (0.649–0.877)
Breakfast skipping	1	0.861	1	0.670
Meals with company	3	0.824 (0.791–0.888)	3	0.802 (0.744–0.839)
Perception of body weight	1	0.878	1	0.892
Nutrition knowledge	1	0.763	1	0.840

#### Reliability of physical activity and sedentary behaviors questionnaires

The range of ICCs for physical activity and sedentary behaviors questions was 0.125–0.935 in adults and 0.001–0.996 in children (Table 2 and Additional files 3 and 4: Tables S3 and S4). A disproportionally low value of ICC was observed in the children’s questionnaire for the question “*On weekdays, how many days did your child walk for other transportation purposes?” (not including walking to and/or from school)*. Overall, in adults 50.0% of ICCs were ranked as “excellent”, 36.1% as “good”, 5.6% as “moderate” and 8.3% at poor. In children, 70.6% of ICCs were ranked as “excellent”, 20.6% as “good”, 2.9% as “moderate” and 5.9% as “poor”. In adults, the lower ICCs values were observed for the questions related to type and frequency of physical activity, while no substantial differences were observed in ICCs values among categories of questions.

**Table 2:** Summary of reliability measures of physical activity and sedentary behaviors questions in adults and children

	Number of questions	Mean ICC (range)
Adults		
Type and frequency of physical activity	6	0.518 (0.306–0.733)
Type and frequency of sedentary behaviors	5	0.820 (0.584–0.935)
Social support for physical activity	3	0.782 (0.737–0.821)
Inactivity perceptions/attitudes related to:		
Lack of time/interest/motivation/enjoyment	5	0.817 (0.732–0.881)
Body image and health	3	0.735 (0.673–0.777)
Environmental factors	6	0.673 (0.125–0.826)
Self-efficacy of physical activity engagement	7	0.848 (0.705–0.910)
Physical activity recommendations knowledge	1	0.796
Children		
Type and frequency of physical activity	20	0.797 (0.001–0.996)
Type and frequency of sedentary behaviors	5	0.773 (0.692–0.838)
Parental support for physical activity	2	0.847 (0.805–0.888)
Parental sabotage for physical activity	2	0.766 (0.707–0.824)
Inactivity perceptions/attitudes	4	0.803 (0.772–0.835)
Physical activity recommendations knowledge	1	0.852

#### Reliability of family’s energy balance related behaviors questionnaire

ICCs in the questions of the family’s energy balance related behaviors ranged from 0.088 to 0.887 (Table 3 and Additional file 5: Table S5). The question regarding breakfast consumption on weekend days by the child had the lowest ICC. 13.9% of ICCs were ranked as “excellent”, 62.5% as “good”, 13.9% as “moderate” and 9.7% as poor. Similar reliability was observed among categories of questions. With the exception of the questions of screen activities, ICCs for the questions regarding the child were relatively lower, compared to those regarding the parent/caregiver.

**Table 3:** Summary of reliability measures of family’s energy balance behaviors questions

	Number of questions	Mean ICC (range)
Frequency of consumption of selected food groups		
Parent	10	0.690 (0.480–0.827)
Child	9	0.633 (0.371–0.822)
Breakfast consumption		
Parent	2	0.616 (0.457–0.775)
Child	2	0.185 (0.088–0.281)
Consumption of selected foods/food groups at breakfast		
Parent	8	0.620 (0.367–0.814)
Child	8	0.725 (0.617–0.810)
Physical activity levels		
Parent	2	0.635 (0.569–0.700)
Child	2	0.494 (0.367–0.620)
Screen activities		
Parent	2	0.502 (0.327–0.676)
Child	2	0.671 (0.647–0.694)
Availability of foods at home	8	0.7200 (0.625–0.794)
Family habits related to diet	3	0.695 (0.563–0.793)
Family habits related to physical activity/sedentary behaviors	6	0.713 (0.517–0.825)
Perceptions about health	3	0.653 (0.570–0.727)
Availability of electronic devices at child’s room	5	0.792 (0.700–0.887)

## Discussion

Despite substantial advances in nutritional epidemiology, assessment of diet and other lifestyle factors, such as physical activity remains a growing field, as respective tools need to capture multiple aspects of nutrition and overall lifestyle. In the present manuscript we described the development of five questionnaires, one for dietary habits in adults and children, one for physical activity in adults and children and one for overall family’s energy balance-related behaviors. We focused not only on the single assessment of dietary intake and physical activity levels, but also on the behaviors towards them, thus evaluating barriers and/or facilitators of a healthy (or unhealthy) lifestyle. Results from our reliability study suggest that the developed tools have at least good reliability and thus may be used for evaluation of the effectiveness of interventions at the community level, such as the Feel4Diabetes study.

The tools for the assessment of both diet and physical activity are commonly characterized by moderate reproducibility. Our reliability results in the food frequency and eating behaviors questionnaire were similar with those reported to other food frequency questionnaires, in both adults [19, 20] and children [21–24]. Similarly, the physical activity and sedentary behaviors questionnaire had comparable reliability with other physical activity questionnaires for adults [17, 25, 26] and children [10]. Of note, in both questionnaires, questions regarding attitudes and perceptions, social support or knowledge did not show higher reliability, compared to questions regarding dietary or physical activity habits. Therefore, the accurate reporting of such aspects of lifestyle should not be taken for granted and the reproducibility of such questions should also be confirmed by reliability studies. This observation is of high importance in behavioral interventions, as it may confound the evaluation of their effectiveness.

When comparing the developed questionnaires, overall reliability was lower in the family’s energy balance-related behaviors questionnaire, compared to the other two questionnaires. This is an expected finding, due to the nature of the questions included in this questionnaire: most of the questions were wide-ranging and qualitative and thus a definite and reproducible answer was more difficult to be obtained. A lower reliability does not necessarily diminish its value, but may limit its use. In fact, this questionnaire was developed as a tool to assess the school-based intervention of the Feel4Diabetes study, and not the intervention on high-risk families. As such, it may be applied in large populations for the detection of individuals at risk of poor health behaviors, where a high sensitivity is not required.

The developed questionnaires have several strengths. They have been designed for a multi-country population, under standardized procedures, including reliability studies in various populations, with different socio-economic background, thus making them applicable for various populations. They also provide a holistic approach of diet and physical activity, exploring various aspects regarding their determinants. On the other hand, they are subjected to inherent limitations that are common in the assessment of lifestyle parameters. Even if we overcome the constraints on precision, self-report bias due to social desirability and approval [27] or intervention-associated bias [28] may compromise the accuracy of the information obtained. In addition, the developed questionnaires have been tested for their reliability in European countries and thus their application in other populations may require additional validity studies. Nevertheless, the use of questionnaires still remains the only choice when it comes to the evaluation of lifestyle in large population groups and their use may provide valuable information that should always be interpreted keeping in mind their limitations.

## Conclusions

The developed questionnaires for adults and school-aged children are valid tools for the assessment of dietary intake, physical activity and overall energy balance-related behaviors in the context of the Feel4Diabetes intervention, as well as any other similar community based intervention.

## Supplementary information

**Additional file 1: Table S1** Intra-class correlation coefficients for test-retest in questions of the food- frequency and eating behaviors questionnaire for adults.

**Additional file 2: Table S2** Intra-class correlation coefficients for test-retest in questions of the food-frequency and eating behaviors questionnaire for children.

**Additional file 3: Table S3** Intra-class correlation coefficients for test-retest in questions of the physical activity and sedentary behaviors questionnaire for adults.

**Additional file 4: Table S4** Intra-class correlation coefficients for test-retest in questions of the physical activity and sedentary behaviors questionnaire for children.

**Additional file 5: Table S5** Intra-class correlation coefficients for test-retest in questions of the family’s energy balance related behaviors questionnaire.

## Data Availability

The datasets used and/or analysed during the current study are available from the corresponding author on reasonable request.

## References

[1] Hemmingsen B, Gimenez-Perez G, Mauricio D, Roque IFM, Metzendorf MI, Richter B (2017). Diet, physical activity or both for prevention or delay of type 2 diabetes mellitus and its associated complications in people at increased risk of developing type 2 diabetes mellitus. Cochrane Database Syst Rev.

[2] Van Hulst A, Paradis G, Harnois-Leblanc S, Benedetti A, Drapeau V, Henderson M (2018). Lowering saturated fat and increasing vegetable and fruit intake may increase insulin sensitivity 2 years later in children with a family history of obesity. J Nutr.

[3] Henderson M, Benedetti A, Barnett TA, Mathieu ME, Deladoey J, Gray-Donald K (2016). Influence of adiposity, physical activity, fitness, and screen time on insulin dynamics over 2 years in children. JAMA Pediatr.

[4] Magkos F, Yannakoulia M (2003). Methodology of dietary assessment in athletes: concepts and pitfalls. Curr Opin Clin Nutr Metab Care.

[5] Serra-Majem L, Frost Andersen L, Henrique-Sanchez P, Doreste-Alonso J, Sanchez-Villegas A, Ortiz-Andrelluchi A (2009). Evaluating the quality of dietary intake validation studies. Br J Nutr.

[6] Ortiz-Andrellucchi A, Henriquez-Sanchez P, Sanchez-Villegas A, Pena-Quintana L, Mendez M, Serra-Majem L (2009). Dietary assessment methods for micronutrient intake in infants, children and adolescents: a systematic review. Br J Nutr.

[7] Jacques PF, Tucker KL (2001). Are dietary patterns useful for understanding the role of diet in chronic disease?. Am J Clin Nutr.

[8] Cullen KW, Watson K, Zakeri I (2008). Relative reliability and validity of the block kids questionnaire among youth aged 10 to 17 years. J Am Diet Assoc.

[9] Silsbury Z, Goldsmith R, Rushton A (2015). Systematic review of the measurement properties of self-report physical activity questionnaires in healthy adult populations. BMJ Open.

[10] Benitez-Porres J, Lopez-Fernandez I, Raya JF, Alvarez Carnero S, Alvero-Cruz JR, Alvarez CE (2016). Reliability and validity of the PAQ-C questionnaire to assess physical activity in children. J School Health.

[11] Ball K, Timperio AF, Crawford DA (2006). Understanding environmental influences on nutrition and physical activity behaviors: where should we look and what should we count?. Int J Behav Nutr Phys Act.

[12] Mawditt C, Sacker A, Britton A, Kelly Y, Cable N (2016). The clustering of health-related behaviours in a British population sample: testing for cohort differences. Prev Med.

[13] Stiglic N, Viner RM (2019). Effects of screentime on the health and well-being of children and adolescents: a systematic review of reviews. BMJ Open.

[14] Mouratidou T, Miguel ML, Androutsos O, Manios Y, De Bourdeaudhuij I, Cardon G (2014). Tools, harmonization and standardization procedures of the impact and outcome evaluation indices obtained during a kindergarten-based, family-involved intervention to prevent obesity in early childhood: the ToyBox-study. Obes Rev.

[15] Sousa VD, Rojjanasrirat W (2011). Translation, adaptation and validation of instruments or scales for use in cross-cultural health care research: a clear and user-friendly guideline. J Eval Clin Pract.

[16] Hemio K, Polonen A, Ahonen K, Kosola M, Viitasalo K, Lindstrom J (2014). A simple tool for diet evaluation in primary health care: validation of a 16-item food intake questionnaire. Int J Environ Res Public Health.

[17] Craig CL, Marshall AL, Sjostrom M, Bauman AE, Booth ML, Ainsworth BE (2003). International physical activity questionnaire: 12-country reliability and validity. Med Sci Sports Exerc.

[18] Manios Y, Androutsos O, Katsarou C, Iotova V, Socha P, Geyer C (2014). Designing and implementing a kindergarten-based, family-involved intervention to prevent obesity in early childhood: the ToyBox-study. Obes Rev : Official J Int Association Study of Obes.

[19] Bohlscheid-Thomas S, Hoting I, Boeing H, Wahrendorf J (1997). Reproducibility and relative validity of food group intake in a food frequency questionnaire developed for the German part of the EPIC project. Eur Prospective Invest Cancer Nutr Int J Epidemiol.

[20] Marventano S, Mistretta A, Platania A, Galvano F, Grosso G (2016). Reliability and relative validity of a food frequency questionnaire for Italian adults living in Sicily, southern Italy. Int J Food Sci Nutr.

[21] Gwynn JD, Flood VM, D'Este CA, Attia JR, Turner N, Cochrane J (2011). The reliability and validity of a short FFQ among Australian aboriginal and Torres Strait islander and non-indigenous rural children. Public Health Nutr.

[22] Neuhouser ML, Lilley S, Lund A, Johnson DB (2009). Development and validation of a beverage and snack questionnaire for use in evaluation of school nutrition policies. J Am Diet Assoc.

[23] Vereecken CA, Maes L (2003). A Belgian study on the reliability and relative validity of the health behaviour in school-aged children food-frequency questionnaire. Public Health Nutr.

[24] Poulain Tanja, Spielau Ulrike, Vogel Mandy, Körner Antje, Kiess Wieland (2019). CoCu: A new short questionnaire to evaluate diet composition and culture of eating in children and adolescents. Clinical Nutrition.

[25] Riviere F, Widad FZ, Speyer E, Erpelding ML, Escalon H, Vuillemin A (2018). Reliability and validity of the French version of the global physical activity questionnaire. J Sport Health Sci.

[26] Busschaert C, De Bourdeaudhuij I, Van Holle V, Chastin SF, Cardon G, De Cocker K (2015). Reliability and validity of three questionnaires measuring context-specific sedentary behaviour and associated correlates in adolescents, adults and older adults. Int J Behav Nutr Phys Act.

[27] Hebert JR, Clemow L, Pbert L, Ockene IS, Ockene JK (1995). Social desirability bias in dietary self-report may compromise the validity of dietary intake measures. Int J Epidemiol.

[28] Natarajan L, Pu M, Fan J, Levine RA, Patterson RE, Thomson CA (2010). Measurement error of dietary self-report in intervention trials. Am J Epidemiol.

