# Supplementary material for: Development and reliability of questionnaires for the assessment of diet and physical activity behaviors in a multi-country sample in Europe the Feel4Diabetes Study
Source: BMC Endocr Disord. 2020 Mar 12;20(Suppl 1):135. doi: 10.1186/s12902-019-0469-x (PMC7066729; doi:10.1186/s12902-019-0469-x)
Supplement: Supplementary file 1 — Additional file 1: Table S1 Intra-class correlation coefficients for test-retest in questions of the food- frequency and eating behaviors questionnaire for adults. [file 12902_2019_469_MOESM1_ESM.docx]

## Table S1

Intra-class correlation coefficients for test-retest in questions of the food- frequency and eating behaviors questionnaire for adults.

| **Questions** | **ICC** | **CI** | |
| --- | --- | --- | --- |
|  |  | **Lower** | **Upper** |
| How often do you consume the following main meals on weekdays: | | | |
| breakfast | 0.905 | 0.870 | 0.931 |
| lunch | 0.712 | 0.601 | 0.792 |
| dinner | 0.877 | 0.830 | 0.912 |
| How often do you consume the following main meals on weekend days: | | | |
| breakfast | 0.876 | 0.828 | 0.911 |
| lunch | 0.850 | 0.791 | 0.893 |
| dinner | 0.811 | 0.737 | 0.865 |
| How often do you consume the following snacks on weekdays: | | | |
| morning snack | 0.956 | 0.938 | 0.969 |
| afternoon snack | 0.882 | 0.835 | 0.916 |
| evening snack | 0.922 | 0.890 | 0.945 |
| other snacks | 0.842 | 0.775 | 0.889 |
| How often do you consume the following snacks on weekend days: | | | |
| morning snack | 0.238 | -0.084 | 0.564 |
| afternoon snack | 0.838 | 0.771 | 0.886 |
| evening snack | 0.914 | 0.878 | 0.939 |
| other snacks | 0.849 | 0.781 | 0.896 |
| How often do you consume the following foods/ food groups in your BREAKFAST: | | | |
| Fruits and berries | 0.839 | 0.772 | 0.886 |
| Vegetables | 0.861 | 0.804 | 0.902 |
| Low-fibre breakfast cereals (e.g. corn flakes or rice crispies, coco pops) | 0.667 | 0.527 | 0.766 |
| Whole grain breakfast cereal, müsli | 0.826 | 0.753 | 0.877 |
| White bread, tortillia, melba toast, rusk, etc. | 0.843 | 0.766 | 0.89 |
| Whole grain bread, tortillia, melba toast, rusk, etc. | 0.852 | 0.790 | 0.896 |
| Milk or milk products, unsweetened (e.g. natural yogurt) | 0.801 | 0.718 | 0.859 |
| Milk or milk products, sweetened (e.g. yogurt, chocolate milk) | 0.881 | 0.832 | 0.916 |
| Cheese | 0.918 | 0.884 | 0.942 |
| Meat or meat products (e.g. cold cuts, bacon, sausages) | 0.881 | 0.831 | 0.916 |
| Sweet or salty pastries (e.g. pancake, cookie, cake, croissant, cheese pie) | 0.845 | 0.781 | 0.89 |
| Eggs (boiled, fried, scrambled, omelet) | 0.828 | 0.757 | 0.878 |
| Water | 0.852 | 0.79 | 0.895 |
| Soft drinks and juices containing sugar | 0.852 | 0.789 | 0.896 |
| Coffee | 0.924 | 0.893 | 0.946 |
| Tea | 0.908 | 0.870 | 0.935 |
| What is the main reason that you usually skip breakfast? | 0.861 | 0.806 | 0.900 |
| How often do you have the following meals with others, with family, friends or colleagues: | | | |
| breakfast | 0.888 | 0.846 | 0.919 |
| lunch | 0.793 | 0.714 | 0.851 |
| dinner | 0.791 | 0.711 | 0.849 |
| How many servings of raw or cooked vegetables do you eat? | 0.782 | 0.699 | 0.842 |
| How many servings of legumes do you eat (e.g. lentils, beans, peas)? | 0.877 | 0.830 | 0.911 |
| How many servings of fruits or berries do you eat? | 0.87 | 0.820 | 0.906 |
| How much bread and other cereals do you eat per day: | | | |
| slice(s) of rye- or crispbread (more than10 g fibre/100g) | 0.625 | 0.468 | 0.736 |
| slice(s) of graham or mixed grain bread (4-10 g fibre/100g) | 0.789 | 0.701 | 0.851 |
| slice(s) of white bread (less than 4 g fibre/100g) | 0.758 | 0.656 | 0.829 |
| cup(s) of porridge (e.g. rye, oat or wheat flake porridge) | 0.349 | 0.069 | 0.545 |
| cup(s) of low-fibre breakfast cereals (e.g. corn flakes or rice crispies) | 0.791 | 0.702 | 0.854 |
| cup(s) of muesli or high-fibre breakfast cereals | 0.874 | 0.82 | 0.912 |
| cup(s) of whole-grain pasta or rice | 0.719 | 0.597 | 0.804 |
| cup(s) of regular pasta or rice | 0.771 | 0.674 | 0.839 |
| How many servings of sweets, biscuits, ice cream, cakes, pastries do you eat? | 0.873 | 0.824 | 0.908 |
| How many servings of salty snacks/fast food do you eat? | 0.734 | 0.631 | 0.808 |
| How many servings of nuts or seeds do you eat? | 0.879 | 0.832 | 0.912 |
| How often do you consume the following fats in your raw or boiled vegetables (consumed as salad): | | | |
| olive oil | 0.89 | 0.845 | 0.921 |
| rapeseed oil | 0.88 | 0.830 | 0.916 |
| other vegetable oil (e.g. sunflower oil) | 0.845 | 0.780 | 0.891 |
| margarine | 0.846 | 0.781 | 0.892 |
| butter | 0.873 | 0.821 | 0.91 |
| cream, sour cream | 0.841 | 0.774 | 0.888 |
| mayonnaise, French dressing etc | 0.786 | 0.696 | 0.849 |
| How often do you consume the following fats with your cooked dish: | | | |
| olive oil | 0.907 | 0.868 | 0.934 |
| rapeseed oil | 0.936 | 0.908 | 0.955 |
| other vegetable oil (e.g. sunflower oil) | 0.892 | 0.846 | 0.924 |
| margarine | 0.786 | 0.693 | 0.851 |
| butter | 0.853 | 0.791 | 0.897 |
| cream, sour cream | 0.854 | 0.792 | 0.898 |
| mayonnaise, French dressing etc | 0.883 | 0.834 | 0.918 |
| What kind of fat spread do you usually use on or with your bread? | 0.855 | 0.800 | 0.895 |
| How many servings of unsweetened milk and milk products (e.g. milk, natural yoghurt) do you consume per week: | | | |
| servings of low/free fat (less than 2 % fat) milk/milk product | 0.889 | 0.845 | 0.921 |
| servings of full fat (equal or more than2 % fat) milk/milk product | 0.908 | 0.870 | 0.935 |
| How many servings of sugared milk products (e.g. chocolate milk, yoghurt) do you consume per week: | | | |
| servings of low/free fat (less than 2 % fat) milk/milk product | 0.713 | 0.596 | 0.796 |
| servings of full fat (equal or more than2 % fat) milk/milk product | 0.924 | 0.893 | 0.946 |
| How much cheese do you eat per week? Count also cheese consumed as food ingredient or side dish: | | | |
| (servings) of reduced-fat cheese less than 20% | 0.873 | 0.822 | 0.912 |
| (servings) of regular fat cheese equal or more than 20% | 0.878 | 0.829 | 0.913 |
| How many servings of red meat (e.g. pork, beef, veal, lamb) or processed meat (e.g. bacon, hamburger or sausages) do you eat? | 0.855 | 0.799 | 0.895 |
| How many servings of white meat (e.g. poultry, rabbit) do you eat? | 0.918 | 0.887 | 0.941 |
| How many servings of fish and seafood do you eat? | 0.918 | 0.886 | 0.94 |
| How much of the following beverages do you drink per week: | | | |
| glass(es) of water (1 glass=250 mL) | 0.878 | 0.828 | 0.913 |
| cup(s) of tea (1 cup=250 mL) | 0.921 | 0.889 | 0.943 |
| cup(s) of coffee (1 cup=250 mL) | 0.930 | 0.902 | 0.950 |
| glass(es) of soft drink with sugar (1 glass = 250 mL) | 0.803 | 0.723 | 0.86 |
| glass(es) of soft drink without sugar, e.g. Coca Cola Light (1 glass = 250 mL) | 0.727 | 0.616 | 0.806 |
| glass(es) of fruit juice freshly squeezed or pre-packed without sugar (1 glass = 250 mL) | 0.947 | 0.925 | 0.962 |
| glass(es) of juice containing sugar (1 glass =250 mL) | 0.915 | 0.880 | 0.940 |
| glass(es) of beer/cider (1 beer glass = 330 mL) | 0.943 | 0.920 | 0.960 |
| glass(es) of wine (1 wine glass = 125 mL) | 0.904 | 0.864 | 0.932 |
| glass(es) other spirits (1 glass = 40 mL) | 0.916 | 0.871 | 0.946 |
| What is your opinion about your current body weight? | 0.878 | 0.831 | 0.912 |
| In your opinion, what is the minimum recommended consumption of fruits, berries and vegetables for adults per day? | 0.763 | 0.673 | 0.829 |
