# Supplementary material for: Development and reliability of questionnaires for the assessment of diet and physical activity behaviors in a multi-country sample in Europe the Feel4Diabetes Study
Source: BMC Endocr Disord. 2020 Mar 12;20(Suppl 1):135. doi: 10.1186/s12902-019-0469-x (PMC7066729; doi:10.1186/s12902-019-0469-x)
Supplement: Supplementary file 2 — Additional file 2: Table S2 Intra-class correlation coefficients for test-retest in questions of the food-frequency and eating behaviors questionnaire for children. [file 12902_2019_469_MOESM2_ESM.docx]

## Table S2

Intra-class correlation coefficients for test-retest in questions of the food-frequency and eating behaviors questionnaire for children.

| **Questions** | **ICC** | | **CI** | |
| --- | --- | --- | --- | --- |
|  |  |  | **Lower** | **Upper** |
| How often does your child consume the following main meals on weekdays: | | | | |
| breakfast | 0.878 | 0.832 | | 0.911 |
| lunch | 0.853 | 0.798 | | 0.893 |
| dinner | 0.869 | 0.819 | | 0.905 |
| How often does your child consume the following main meals on weekend days: | | | | |
| breakfast | 0.778 | 0.694 | | 0.838 |
| lunch | 0.828 | 0.762 | | 0.876 |
| dinner | 0.795 | 0.717 | | 0.852 |
| How often does your child consume the following snacks on weekdays: | | | | |
| morning snack | 0.896 | 0.855 | | 0.925 |
| afternoon snack | 0.887 | 0.843 | | 0.918 |
| evening snack | 0.925 | 0.894 | | 0.947 |
| other snacks | 0.899 | 0.855 | | 0.930 |
| How often does your child consume the following snacks on weekend days: | | | | |
| morning snack | 0.792 | 0.705 | | 0.854 |
| afternoon snack | 0.784 | 0.699 | | 0.845 |
| evening snack | 0.904 | 0.863 | | 0.933 |
| other snacks | 0.819 | 0.741 | | 0.874 |
| How often does your child consume the following foods/ food groups in his/her BREAKFAST: | | | | |
| Fruits and berries | 0.727 | 0.621 | | 0.803 |
| Vegetables | 0.702 | 0.586 | | 0.786 |
| Low-fibre breakfast cereals (e.g. corn flakes or rice crispies, coco pops) | 0.837 | 0.773 | | 0.882 |
| Whole grain breakfast cereal, müsli | 0.814 | 0.741 | | 0.867 |
| White bread, tortillia, melba toast, rusk, etc. | 0.701 | 0.583 | | 0.785 |
| Whole grain bread, tortillia, melba toast, rusk, etc. | 0.791 | 0.706 | | 0.851 |
| Milk or milk products, unsweetened (e.g. natural yogurt) | 0.649 | 0.513 | | 0.747 |
| Milk or milk products, sweetened (e.g. yogurt, chocolate milk) | 0.851 | 0.793 | | 0.892 |
| Cheese | 0.838 | 0.774 | | 0.884 |
| Meat or meat products (e.g. cold cuts, bacon, sausages) | 0.839 | 0.777 | | 0.884 |
| Sweet or salty pastries (e.g. pancake, cookie, cake, croissant, cheese pie) | 0.877 | 0.829 | | 0.911 |
| Eggs (boiled, fried, scrambled, omelet) | 0.69 | 0.571 | | 0.776 |
| Water | 0.78 | 0.697 | | 0.841 |
| Soft drinks and juices containing sugar | 0.875 | 0.825 | | 0.91 |
| Tea | 0.863 | 0.810 | | 0.901 |
| What is the main reason that your child usually skips breakfast? | 0.674 | 0.550 | | 0.763 |
| How often does your child have the following meals with others, with at least one parent: | | | | |
| breakfast | 0.823 | 0.757 | | 0.872 |
| lunch | 0.744 | 0.647 | | 0.815 |
| dinner | 0.839 | 0.778 | | 0.883 |
| How many servings of raw or cooked vegetables does your child eat? | 0.758 | 0.667 | | 0.824 |
| How many servings of legumes does your child eat (e.g. lentils, beans, peas)? | 0.830 | 0.767 | | 0.877 |
| How many servings of fruits or berries does your child eat? | 0.820 | 0.751 | | 0.87 |
| How much bread and other cereals does your child eat per day: | | | | |
| slice (s) of rye- or crispbread (more than10 g fibre/100g) | 0.396 | 0.139 | | 0.577 |
| slice(s) of graham or mixed grain bread (4-10 g fibre/100g) | 0.719 | 0.602 | | 0.802 |
| slice(s) of white bread (less than 4 g fibre/100g) | 0.001 | -0.406 | | 0.29 |
| cup(s) of porridge (e.g. rye, oat or wheat flake porridge) | 0.820 | 0.742 | | 0.875 |
| cup(s) of low-fibre breakfast cereals (e.g. corn flakes or rice crispies) | 0.814 | 0.734 | | 0.87 |
| cup(s) of muesli or high-fibre breakfast cereals | 0.426 | 0.179 | | 0.599 |
| cup(s) of whole-grain pasta or rice | 0.669 | 0.526 | | 0.768 |
| cup(s) of regular pasta or rice | 0.760 | 0.660 | | 0.831 |
| How many servings of sweets, biscuits, ice cream, cakes, pastries does your child eat? | 0.838 | 0.777 | | 0.883 |
| How many servings of salty snacks/fast food does your child eat? | 0.765 | 0.676 | | 0.83 |
| How many servings of nuts or seeds does your child eat? | 0.798 | 0.722 | | 0.854 |
| How often does your child consume the following fats in his/her raw or boiled vegetables (consumed as salad): | | | | |
| Olive oil | 0.863 | 0.809 | | 0.902 |
| Rapeseed oil | 0.939 | 0.914 | | 0.957 |
| Other vegetable oil (e.g. sunflower oil) | 0.700 | 0.580 | | 0.786 |
| Margarine | 0.484 | 0.272 | | 0.634 |
| Butter | 0.867 | 0.814 | | 0.905 |
| Cream, sour cream | 0.906 | 0.869 | | 0.933 |
| Mayonnaise, French dressing etc | 0.665 | 0.533 | | 0.76 |
| How often does your child consume the following fats in his/her cooked dish: | | | | |
| Olive oil | 0.849 | 0.789 | | 0.892 |
| Rapeseed oil | 0.843 | 0.779 | | 0.889 |
| Other vegetable oil (e.g. sunflower oil) | 0.843 | 0.779 | | 0.888 |
| Margarine | 0.713 | 0.593 | | 0.798 |
| Butter | 0.880 | 0.831 | | 0.915 |
| Cream, sour cream | 0.864 | 0.808 | | 0.904 |
| Mayonnaise, French dressing etc | 0.817 | 0.742 | | 0.87 |
| What kind of fat spread does your child usually use on or with his/her bread? | 0.868 | 0.817 | | 0.905 |
| How many servings of unsweetened milk and milk products (e.g. milk, natural yoghurt) does your child consume per week: | | | | |
| servings of low/free fat (less than 2 % fat) milk/milk product | 0.953 | 0.933 | | 0.966 |
| servings of full fat (equal or more than2 % fat) milk/milk product | 0.878 | 0.829 | | 0.914 |
| How many servings of sugared milk products (e.g. chocolate milk, yoghurt) does your child consume per week: | | | | |
| servings of low/free fat (less than 2 % fat) milk/milk product | 0.879 | 0.83 | | 0.914 |
| servings of full fat (equal or more than2 % fat) milk/milk product | 0.818 | 0.744 | | 0.871 |
| How much cheese does your child eat per week? Count also cheese consumed as food ingredient or side dish: | | | | |
| (servings) of reduced-fat cheese less than 20% | 0.772 | 0.676 | | 0.838 |
| (servings) of regular fat cheese equal or more than 20% | 0.811 | 0.736 | | 0.865 |
| How many servings of red meat (e.g. pork, beef, veal, lamb) or processed meat (e.g. bacon, hamburger or sausages) does your child eat? | 0.808 | 0.734 | | 0.861 |
| How many servings of white meat (e.g. poultry, rabbit) does your child eat? | 0.805 | 0.730 | | 0.859 |
| How many servings of fish and seafood does your child eats? | 0.909 | 0.874 | | 0.934 |
| How much of the following beverages does your child drink per week: | | | | |
| glass(es) of water (1 glass=250 mL) | 0.884 | 0.837 | | 0.918 |
| cup(s) of tea (1 cup=250 mL) | 0.855 | 0.796 | | 0.897 |
| glass(es) of soft drink with sugar (1 glass = 250 mL) | 0.855 | 0.796 | | 0.898 |
| glass(es) of soft drink without sugar, e.g. Coca Cola Light (1 glass = 250 mL) | 0.808 | 0.730 | | 0.864 |
| glass(es) of fruit juice freshly squeezed or pre-packed without sugar (1 glass = 250 mL) | 0.687 | 0.560 | | 0.778 |
| glass(es) of juice containing sugar (1 glass =250 mL) | 0.349 | 0.036 | | 0.56 |
| What is your opinion about your child's body weight? | 0.892 | 0.851 | | 0.923 |
| In your opinion, what is the minimum recommended consumption of fruits, berries and vegetables for children per day? | 0.840 | 0.778 | | 0.884 |
