# Supplementary material for: Development and reliability of questionnaires for the assessment of diet and physical activity behaviors in a multi-country sample in Europe the Feel4Diabetes Study
Source: BMC Endocr Disord. 2020 Mar 12;20(Suppl 1):135. doi: 10.1186/s12902-019-0469-x (PMC7066729; doi:10.1186/s12902-019-0469-x)
Supplement: Supplementary file 3 — Additional file 3: Table S3 Intra-class correlation coefficients for test-retest in questions of the physical activity and sedentary behaviors questionnaire for adults. [file 12902_2019_469_MOESM3_ESM.docx]

## Table S3

Intra-class correlation coefficients for test-retest in questions of the physical activity and sedentary behaviors questionnaire for adults.

| **Questions** | **ICC** | **CI** | |
| --- | --- | --- | --- |
|  |  | **Lower** | **Upper** |
| During the last 7 days, on how many days did you do vigorous physical activities like heavy lifting, digging, aerobics, or fast bicycling? | 0.467 | 0.250 | 0.622 |
| How much time did you usually spend doing vigorous physical activities on one of those days? | 0.733 | 0.608 | 0.818 |
| During the last 7 days, on how many days did you do moderate physical activities like carrying light loads, bicycling at a regular pace, or doubles tennis? | 0.377 | 0.120 | 0.560 |
| How much time did you usually spend doing moderate physical activities on one of those days? | 0.607 | 0.420 | 0.733 |
| During the last 7 days, on how many days did you walk for at least 10 minutes at a time? | 0.618 | 0.467 | 0.727 |
| How much time did you usually spend walking on one of those days? | 0.306 | 0.021 | 0.508 |
| During the last 7 days, how much time did you spend sitting on a week day? | 0.584 | 0.419 | 0.702 |
| How much time do you spend watching TV per day: on weekdays? | 0.879 | 0.833 | 0.913 |
| How much time do you spend watching TV per day: on weekend days? | | | |
| How much time do you spend using a computer, tablet, smartphone per day: on weekdays? | 0.878 | 0.832 | 0.912 |
| How much time do you spend using a computer, tablet, smartphone per day: on weekend days? | 0.824 | 0.756 | 0.874 |
| How often does a member of your household, a friend or a colleague encourage you to: participate to a physical activity? | 0.821 | 0.751 | 0.871 |
| How often does a member of your household, a friend or a colleague encourage you to: walk/ bicycle? | 0.737 | 0.635 | 0.811 |
| How likely are you to say: | | | |
| “I don’t exercise because I don’t have any spare time"? | 0.840 | 0.777 | 0.885 |
| “I don’t exercise because I have other interesting things to do"? | 0.732 | 0.626 | 0.809 |
| “I don’t exercise because I am too lazy/ not motivated to be physically active"? | 0.819 | 0.747 | 0.870 |
| “I don’t exercise because my body weight keeps me away from participation in any kind of physical activity"? | 0.754 | 0.657 | 0.823 |
| “I don’t exercise because I am too shy or embarrassed to be physically active with others"? | 0.673 | 0.545 | 0.766 |
| “I don’t exercise because my neighborhood lacks sidewalks, bicycle lanes, parks or pavements appropriate for walking"? | 0.125 | -0.221 | 0.372 |
| “I don’t exercise because I have to look after my children in the afternoon or in weekends"? | 0.811 | 0.736 | 0.864 |
| “I don’t exercise because my health is poor and I am afraid that I might get injured or damage my health"? | 0.777 | 0.690 | 0.840 |
| “I don’t exercise because I can’t afford it"? | 0.826 | 0.757 | 0.876 |
| “I don’t exercise because there is no one to exercise with"? | 0.788 | 0.704 | 0.848 |
| “I don’t exercise because there are no suitable facilities nearby"? | 0.819 | 0.747 | 0.871 |
| “I don’t exercise because my neighborhood lacks aesthetics and pleasantness to walk or exercise"? | 0.814 | 0.741 | 0.867 |
| “I don’t exercise because my neighborhood is not safe"? | 0.666 | 0.535 | 0.760 |
| “I don’t exercise because of weather conditions"? | 0.785 | 0.700 | 0.846 |
| “I don’t exercise because I don’t enjoy physical activity"? | 0.881 | 0.834 | 0.914 |
| “I don’t exercise because I would never keep up an exercise work out"? | 0.705 | 0.588 | 0.788 |
| How confident do you feel that you can: |  |  |  |
| do physical activity on your own? | 0.910 | 0.876 | 0.936 |
| include physical activity in your daily routine? | 0.820 | 0.750 | 0.871 |
| be physically active during vacation? | 0.890 | 0.847 | 0.921 |
| continue being physically active even when your personal and family responsibilities are more demanding than usual? | 0.836 | 0.771 | 0.882 |
| continue being physically active even when you feel depressed? | 0.900 | 0.861 | 0.928 |
| continue being physically active even when you feel anxious? | 0.876 | 0.826 | 0.911 |
| In your opinion, what are the minimum recommendations for adults, regarding physical activity (minutes of moderate to vigorous intensity physical activity per day)? | 0.796 | 0.715 | 0.853 |
