# Supplementary material for: Development and reliability of questionnaires for the assessment of diet and physical activity behaviors in a multi-country sample in Europe the Feel4Diabetes Study
Source: BMC Endocr Disord. 2020 Mar 12;20(Suppl 1):135. doi: 10.1186/s12902-019-0469-x (PMC7066729; doi:10.1186/s12902-019-0469-x)
Supplement: Supplementary file 4 — Additional file 4: Table S4 Intra-class correlation coefficients for test-retest in questions of the physical activity and sedentary behaviors questionnaire for children. [file 12902_2019_469_MOESM4_ESM.docx]

## Table S4

Intra-class correlation coefficients for test-retest in questions of the physical activity and sedentary behaviors questionnaire for children.

| **Questions** | **ICC** | **CI** | |
| --- | --- | --- | --- |
|  |  | **Lower** | **Upper** |
| Does your child participate in physical activities on a regular basis (such as football, swimming, volleyball, skating)? | 0.939 | 0.909 | 0.959 |
| Activity 1: type of activity | N/A | N/A | N/A |
| Activity 1: times per week | 0.919 | 0.882 | 0.944 |
| Activity 1: total time per week | 0.996 | 0.994 | 0.997 |
| Activity 2: type of activity | N/A | N/A | N/A |
| Activity 2: times per week | 0.885 | 0.797 | 0.935 |
| Activity 2: total time per week | 0.880 | 0.786 | 0.933 |
| Activity 3: type of activity | N/A | N/A | N/A |
| Activity 3: times per week | 0.858 | 0.642 | 0.944 |
| Activity 3: total time per week | 0.907 | 0.766 | 0.963 |
| On weekdays, how many days did your child … |  |  |  |
| walk to and/or from school: days | 0.929 | 0.900 | 0.949 |
| walk to and/or from school: minutes per day | 0.794 | 0.710 | 0.853 |
| walk for other transportation purposes: days | 0.001 | -0.411 | 0.291 |
| walk for other transportation purposes: minutes per day | 0.592 | 0.424 | 0.711 |
| bicycle to and/or from school: days | 0.836 | 0.764 | 0.886 |
| bicycle to and/or from school: minutes per day | 0.903 | 0.857 | 0.934 |
| bicycle for other transportation purposes: days | 0.977 | 0.966 | 0.984 |
| bicycle for other transportation purposes: minutes per day | 0.369 | 0.073 | 0.570 |
| On weekend days, how many days did your child… |  |  |  |
| walk for other transportation purposes: days | 0.965 | 0.951 | 0.975 |
| walk for other transportation purposes: minutes per day | 0.609 | 0.444 | 0.725 |
| bicycle for other transportation purposes: days | 0.826 | 0.751 | 0.878 |
| bicycle for other transportation purposes: minutes per day | 0.821 | 0.741 | 0.876 |
| How much time does your child spend watching TV per day: on weekdays | 0.810 | 0.737 | 0.863 |
| How much time does your child spend watching TV per day: on weekend days | 0.838 | 0.776 | 0.883 |
| How much time does your child spend using a computer, tablet, smartphone per day: on weekdays | 0.701 | 0.587 | 0.784 |
| How much time do your child spend using a computer, tablet, smartphone per day: on weekend days | 0.826 | 0.760 | 0.874 |
| How often does at least one parent/caregiver encourage your child to engage in active physical activity/play? | 0.692 | 0.575 | 0.777 |
| How often does at least one parent/caregiver… |  |  |  |
| participate in physical activity with the child? | 0.888 | 0.845 | 0.919 |
| bring the child to the place where the child can exercise or be physically active? | 0.805 | 0.731 | 0.859 |
| How much time does at least one of the parents watch television/ videos or play computer/electronic games together with your child per day: weekdays | 0.707 | 0.595 | 0.788 |
| How much time does at least one of the parents/ caregivers watch television/ videos or play computer/electronic games together with your child per day: weekend days | 0.824 | 0.756 | 0.872 |
| How much do you agree with the following statements: |  |  |  |
| My child prefers to watch TV or read a book instead of doing something that demands physical activity. | 0.835 | 0.772 | 0.881 |
| TV viewing and use of other electronic devices (such as computer, tablets, smartphones etc) by children may increase their knowledge or their skills. | 0.772 | 0.686 | 0.835 |
| I prefer that my child concentrates on his/her homework, than participating in physical activity/ sports in his/her free time. | 0.789 | 0.709 | 0.847 |
| My child is allowed to skip physical activity/sport planned sessions (e.g. ballet class or team sport training) whenever he/she wants. | 0.814 | 0.743 | 0.865 |
| What are the minimum recommendations for children, regarding physical activity (minutes of moderate to vigorous intensity physical activity per day)? | 0.852 | 0.795 | 0.893 |

N/A: not applicable
