# Supplementary material for: Development and reliability of questionnaires for the assessment of diet and physical activity behaviors in a multi-country sample in Europe the Feel4Diabetes Study
Source: BMC Endocr Disord. 2020 Mar 12;20(Suppl 1):135. doi: 10.1186/s12902-019-0469-x (PMC7066729; doi:10.1186/s12902-019-0469-x)
Supplement: Supplementary file 5 — Additional file 5: Table S5 Intra-class correlation coefficients for test-retest in questions of the family’s energy balance related behaviors questionnaire. [file 12902_2019_469_MOESM5_ESM.docx]

## Table S5

Intra-class correlation coefficients for test-retest in questions of the family’s energy balance related behaviors questionnaire.

| **Questions** | **ICC** | **CI** | |
| --- | --- | --- | --- |
|  |  | **Lower** | **Upper** |
| Please indicate how often do you consume*: | | | |
| water | 0.609 | 0.456 | 0.719 |
| fruits and berries, fresh or frozen | 0.754 | 0.654 | 0.825 |
| fruit and berries, canned or dried | 0.480 | 0.270 | 0.630 |
| fruit juices, freshly squeezed or pre-packed, no sugar | 0.827 | 0.758 | 0.877 |
| soft drinks and juices containing sugar | 0.599 | 0.436 | 0.714 |
| soft drinks, diet (light) | 0.732 | 0.620 | 0.811 |
| coffee | 0.753 | 0.654 | 0.823 |
| vegetables | | | |
| sweets | 0.770 | 0.679 | 0.835 |
| salty snacks/fast food | 0.590 | 0.426 | 0.708 |
| Please indicate how often does your child consume*: | | | |
| water | 0.720 | 0.606 | 0.801 |
| fruits and berries, fresh or frozen | 0.647 | 0.504 | 0.749 |
| fruit and berries, canned or dried | 0.371 | 0.113 | 0.554 |
| fruit juices, freshly squeezed or prepacked, no sugar | 0.752 | 0.652 | 0.823 |
| soft drinks and juices containing sugar | 0.639 | 0.492 | 0.744 |
| soft drinks, diet (light) | 0.737 | 0.627 | 0.815 |
| vegetables | 0.822 | 0.725 | 0.885 |
| sweets | 0.410 | 0.171 | 0.580 |
| salty snacks/fast food | 0.596 | 0.433 | 0.712 |
| On how many days do you usually eat breakfast: weekdays | 0.775 | 0.687 | 0.838 |
| On how many days do you usually eat breakfast: weekend days | 0.457 | 0.245 | 0.609 |
| On how many days does your child usually eat breakfast: weekdays | 0.281 | 0.000 | 0.482 |
| On how many days does your child usually eat breakfast: weekend days | 0.088 | -0.268 | 0.344 |
| How often do you consume the following foods/ food groups as part of your breakfast: | | | |
| fruits, berries and vegetables | 0.618 | 0.467 | 0.726 |
| freshly squeezed juices or pre-packed without sugar | 0.814 | 0.740 | 0.867 |
| soft drinks and juices containing sugar | 0.393 | 0.148 | 0.568 |
| milk or milk products, unsweetened (e.g. cheese, natural yogurt) | 0.667 | 0.534 | 0.762 |
| milk or milk products, sweetened (e.g. yogurt, pudding, chocolate milk) | 0.740 | 0.634 | 0.815 |
| ..sweet or salty pastries (e.g. pancake, cookie, cake, croissant, cheese pie) | 0.637 | 0.494 | 0.740 |
| low fiber cereal and cereal products (e.g. white bread or rusk any type) refined breakfast cereals, such as coco pops) | 0.367 | 0.118 | 0.545 |
| whole grain cereal and cereal products (e.g. brown bread, porridge) | 0.727 | 0.617 | 0.805 |
| How often does your child consume the following foods/ food groups as part of his/her breakfast: | | | |
| fruits, berries and vegetables | 0.707 | 0.590 | 0.790 |
| freshly squeezed juices or pre-packed without sugar | 0.802 | 0.723 | 0.858 |
| soft drinks and juices containing sugar | 0.644 | 0.502 | 0.746 |
| milk or milk products, unsweetened (e.g. cheese, natural yogurt) | 0.727 | 0.617 | 0.805 |
| milk or milk products, sweetened (e.g. yogurt, pudding, chocolate milk) | 0.810 | 0.734 | 0.865 |
| sweet or salty pastries (e.g. pancake, cookie, cake, croissant, cheese pie) | 0.741 | 0.638 | 0.815 |
| low fiber cereal and cereal products (e.g. white bread or rusk any type, refined breakfast cereals, such as coco pops) | 0.617 | 0.461 | 0.728 |
| whole grain cereal and cereal products (e.g. brown bread, porridge) | 0.754 | 0.656 | 0.825 |
| On how many days during the last week were you physically active for a total of at least 30 minutes per day? | | | |
| Weekdays | 0.700 | 0.582 | 0.784 |
| Weekend days | 0.569 | 0.399 | 0.691 |
| On how many days during the last week was your child physically active for a total of at least 1 hour per day? | | | |
| Weekdays | 0.620 | 0.472 | 0.727 |
| Weekend days | 0.367 | 0.119 | 0.546 |
| About how many hours per day do you usually devote to screen-activities (excluding work)? | | | |
| Weekdays | 0.327 | 0.063 | 0.517 |
| Weekend days | 0.676 | 0.549 | 0.768 |
| About how many hours per day does your child usually devote to screen-activities (excluding school)? | | | |
| Weekdays | 0.647 | 0.508 | 0.747 |
| Weekend days | 0.694 | 0.574 | 0.781 |
| On a weekly basis, how often the following foods are available at your home: | | | |
| Fruits | 0.657 | 0.523 | 0.754 |
| Fruit juices, freshly-squeezed or prepacked without sugar | 0.769 | 0.677 | 0.835 |
| Fruit juices, prepacked, containing sugar | 0.736 | 0.630 | 0.811 |
| Soft drinks containing sugar | 0.740 | 0.638 | 0.813 |
| Soft drinks without sugar | 0.794 | 0.712 | 0.853 |
| Vegetables | 0.625 | 0.478 | 0.731 |
| Sweets, biscuits, ice cream, cakes, pastries | 0.730 | 0.625 | 0.806 |
| Salty snacks (e.g. chips, savory pastries) | 0.709 | 0.593 | 0.791 |
| On a weekly basis, how often do you do the following : | | | |
| Consume fresh fruits with your child | 0.793 | 0.713 | 0.851 |
| Be physically active with your child | 0.718 | 0.609 | 0.797 |
| Watch TV together with your child | 0.825 | 0.756 | 0.874 |
| Allow your child to eat sweets and/or salty snacks whenever he/she asks for | 0.728 | 0.621 | 0.805 |
| Allow your child to watch TV or DVD when he/she wants | 0.743 | 0.642 | 0.815 |
| Allow your child to use the computer, mobile or tablet when he or she wants | 0.802 | 0.724 | 0.857 |
| Reward your child by allowing him/her to watch TV/DVD or use the computer, mobile or tablet | 0.517 | 0.329 | 0.652 |
| Reward your child with sweets, salty snacks (e.g. potato chips) or fast food | 0.563 | 0.392 | 0.686 |
| Reward your child by being physically active together with him/her or by taking him/her to the playground or to the park | 0.675 | 0.549 | 0.766 |
| Do you agree or disagree with the statement: | | | |
| I believe that my health and wellbeing is determined by my destiny. | 0.570 | 0.403 | 0.691 |
| I believe that people have little power on preventing diseases. | 0.663 | 0.533 | 0.757 |
| I chose to eat the food that I like without thinking too much about it. | 0.727 | 0.621 | 0.803 |
| Is the following device available in your child's room: | | | |
| TV? | 0.845 | 0.784 | 0.888 |
| DVD player? | 0.718 | 0.603 | 0.799 |
| game console? | 0.700 | 0.579 | 0.786 |
| computer? | 0.887 | 0.842 | 0.919 |
| tablet or smartphone? | 0.810 | 0.734 | 0.864 |

* appropriate household units of measurement were given for each food item
